# Targeting the Homologous Recombination Pathway in Cancer With a Novel Class of RAD51 Inhibitors

**DOI:** 10.3389/fonc.2022.885186

**Published:** 2022-05-13

**Authors:** Peng Gu, Liting Xue, Chunyan Zhao, Wenjing Li, Zhen Jiang, Aiguo Liu, Tingting Li, Lu Liu, Markus Decker, Xiaoxuan Cheng, Wenqing Yang, Renhong Tang

**Affiliations:** ^1^ State Key Laboratory of Translational Medicine and Innovative Drug Development, Jiangsu Simcere Pharmaceutical Co., Ltd., Nanjing, China; ^2^ High School Sophomore, Hangzhou Foreign Languages School, Hangzhou, China

**Keywords:** Keywords: RAD51, small molecule inhibitor, DNA damage response, homologous recombination, synthetic lethality

## Abstract

Targeting DNA damage response (DDR) pathway has been proposed as an approach for amplifying tumor-specific replicative lesions. RAD51 plays a central role in the DDR process, and thus represents a promising anti-tumor target. We here report the discovery of a series of next generation RAD51 inhibitors that can prevent RAD51 foci formation. The lead compounds dramatically impaired human cancer cell growth, induced cell cycle arrest in S-phase, and resulted in elevated γH2AX. Furthermore, cancer cells became sensitized to chemotherapy and other DDR inhibitors. Dosed either as a single agent or in combination with cisplatin, the compounds significantly inhibited tumor growth *in vivo*. By upregulating ATR-CHK1 signaling, the RAD51 inhibitors increased surface PD-L1 levels in various tumor cells, suggesting a potential combination of RAD51 inhibitors with PD-1/PD-L1 blockade. Overall, our findings provide the preclinical rationale to explore RAD51 inhibitors as monotherapy or in combination with chemotherapy, immunotherapy or DDR-targeting therapy in cancer treatment.

## Introduction

The DNA damage response (DDR) is a complex mechanism for DNA damage detection and repair while unrepaired DNA lesions may result in cell death. Genomic instability caused by dysregulation of DDR is one of the major hallmarks of cancer (1). Targeting the DDR in cancers has therefore garnered much attention in recent years yielding novel therapeutic interventions. Most prominently, poly (ADP-ribose) polymerase (PARP) inhibitors such as olaparib have demonstrated clinical benefit in several human cancers (2). Beyond PARP inhibitors, compounds targeting protein kinases involved in activating ATM, ATR and DNA-PK, are currently in clinical trials for hematologic and solid tumors (3).

Homologous recombination (HR) is a central pathway that repairs DNA double-strand breaks (DSBs) caused by endogenous replication stresses and exogenous agents such as ionizing radiation and genotoxic compounds. The DNA replication stress caused by oncogene activation is an important cause of genomic instability in tumorigenesis (4). The HR repair begins with the recruitment of the MRN complex (Mre11, Rad50 and Nbs1) to the DSB site, followed by ATM recruitment and activation (5). Once activated, ATM orchestrates DSB repair by phosphorylating H2AX on Ser139, referred to as γH2AX, and downstream proteins such as 53BP1 and RAD50. In addition, ATR and DNA-PK kinases are also involved in the DDR by interacting with the DNA binding co-activator complex RPA-ATRIP and XRCC6/XRCC5, respectively. The principle role of RAD51, a homologue of *E. coli* RecA, in the HR pathway is well established (6, 7). The RAD51 recombinase assembles at the resected DNA ends of the DSB to form the nucleoprotein filament. Subsequently, the RAD51 filament searches and invades the homologous region in the sister chromatid to form a displacement loop called a D-loop, followed by gap-filling DNA synthesis and ligation to complete the repair. RAD51 filament formation is controlled by several mediators including BRCA2, RAD52 and RAD51 paralogues (8). Importantly, RAD51 deficient cells lead to genomic instability, as RAD51 depletion in chicken cells resulted in chromosome aberrations and cell lethality (9).

RAD51 overexpression is observed in several human malignancies, including pancreatic adenocarcinoma, non-small-cell lung cancer and breast cancer (10). In addition, it is reported that the overexpression of RAD51 confers resistance to PARP inhibitors in triple negative breast cancer cells (11). Moreover, RAD51 foci formation correlates with resistance to PARP inhibitor in breast cancer patients with germline BRCA mutations (12). As such, RAD51 is emerging as an attractive therapeutic target for restoring synthetic lethality in tumors that have developed resistance to PARP inhibitors. The importance of RAD51 in DNA DSB repair is illustrated by studies showing that increased expression of RAD51 and other HR-associated genes in tumor cells is associated with resistance to radiotherapies or chemotherapies that induce DNA damage (13, 14), implying that targeting RAD51 may improve the efficacy of DNA-damaging agents such as irradiation or chemotherapy. Indeed, there has been intense interest in developing small molecule RAD51 inhibitors. First generation RAD51 inhibitors such as B02, RI-1, RI-2 and IBR2 (15), were limited by a poor potency of growth inhibition, displaying micromolar inhibition concentrations (IC_50_) in cellular assay (16). We thus set out to identify a highly potent RAD51 inhibitor with good clinical development ability. Moreover, small molecules that specifically target RAD51 could be used as a powerful tool to further understand the role of RAD51 in DNA repair and beyond.

Here we describe the identification and characterization of next-generation orally bioavailable inhibitors against RAD51 with antiproliferative activities in both *in vitro* and *in vivo* models. Of note, a patent application disclosing structures of the compounds described herein has been submitted. We show that the inhibitor’s antiproliferative effect can be explained by a mechanism of reduced RAD51 nuclear accumulation and RAD51 degradation by the ubiquitin-proteasome pathway. Oral dosing demonstrated dose-dependent anti-tumor activity and a combination benefit with cisplatin in mice implanted with Daudi xenografts. On the basis of these findings, we propose the preclinical rationale to target RAD51 in Burkitt’s lymphoma patients. In addition, we identify the RAD51 inhibitor as a potential synthetic lethal partner for other DDR inhibitors extending the applicability of our identified compound to other tumor types. Furthermore, our findings suggest that RAD51 inhibition may increase the effectiveness of immunotherapy.

## Materials and Methods

### Cell Lines and Culture

A full list of cell lines, their origins, cell growth media and assay media used in this study can be found in **Supplementary Table 1**. All cells were cultured in 5% CO_2_ humidified atmosphere at 37°C.

### Cellular Thermal Shift Assay

For the cell lysate CETSA experiments, Z138 cells were harvested and washed with PBS supplemented with protease and phosphatase inhibitor tablets. The cell suspensions were freeze-thawed three times using liquid nitrogen. The soluble fraction was separated by centrifugation at 20000g for 20 min at 4°C. Then cell lysate was divided into several aliquots for different compounds or temperatures treatment. After 10-30 min incubation at room temperature (RT) with compounds, cell lysates were heated at indicated temperatures for 3 min followed by cooling for 3 min at RT. The appropriate temperatures were determined in preliminary CETSA experiments (data no shown). The heated lysates were centrifuged at 20000g for 20 min at 4°C and supernatants were transferred to new microtubes and subjected to western blot analysis.

### Immunofluorescence Staining

Cells were seeded on coverslips which were pre-coated with poly-L-lysine (Sigma-Aldrich). After the drug treatment, cells were fixed with 4% paraformaldehyde on ice for 20 min, followed by 3 washes with cold PBS. Fixed cells were permeabilized by 0.25% Triton X-100 for 10 min on ice and blocked by 5% FBS in PBS for 1 hour at RT. For staining, blocking buffer was removed and primary antibodies were diluted in 5% FBS and then incubated with cells overnight at 4°C. Coverslips were washed with PBS for 3 times and then incubated with 5% FBS containing Alexa Fluor^®^ secondary antibodies at RT for 1 hour. After washing with PBS for 3 times, coverslips were mounted on glass slides with anti-fade fluorescence mounting medium. Images were acquired with an inverted fluorescent microscope (Nikon Eclipse Ti2-U) and processed with Adobe Photoshop CC 2018. For RAD51 foci quantification, cells with more than 10 foci were counted as positive and at least 300 cells per experiment were scored for the presence of foci. Each experiment was repeated 3 times independently.

### Immunofluorescence Histochemistry

To measure γH2AX foci formation *in vivo*, after the drug treatment, mice with Daudi xenograft were euthanized and the tumors were dissected and washed briefly with cold PBS, then fixed overnight in 10 ml of fresh neutral buffered formalin (10%). The tissues were gradually dehydrated with 20% and 30% sucrose solutions until tissue sinks. Dehydrated issues were transferred to OCT (Optimum cutting temperature compound) chamber and surround with OCT, dissected into 10um thickness. The slices were fixed 40 min with cool acetone, then equilibrated with PBS for 10 min and blocked by incubation in PBS containing 0.1% Triton X-100, 0.5% Tween-20 (PBSTT) supplemented with 4% (w/v) BSA and 4% goat serum for 2 h at room temperature. For staining, slides were then incubated with rabbit monoclonal anti-γH2AX antibody in PBSTT containing 4% (w/v) BSA and 4% goat serum overnight at 4°C. The sections were washed 3 times for 5 min each in PBSTT and then incubated with anti-rabbit Alexa Fluor^®^ 488 antibody in PBSTT for 2 h at room temperature. Nuclei were counterstained with DAPI in mounting media (Abcam). γH2AX foci were visualized under Nikon ECLIIPSE Ni-U microscope with 40× objective. Images were processed with Adobe Photoshop CC 2018.

### Western Blot

After compounds treatment at indicated time points, cells were washed with PBS and lysed by RIPA lysis buffer (ThermoFisher) supplemented with protease and phosphatase inhibitor tablets. Cell lysate were cleared by centrifugation at 12000g for 10 min. Protein concentration were measured by BCA protein assay kit (ThermoFisher) and equal amount of protein samples were separated by 4–15% Mini-PROTEAN^®^ TGX™ Precast Protein Gels (Bio-Rad) and transferred by Trans-Blot^®^ Turbo™ System (Bio-Rad). Membranes were blocked with 5% non-fat milk or BSA in Tris Buffered Saline with 0.05% Tween-20 (TBS-T) and incubated with primary antibody overnight at 4°C. Membranes were then washed with TBS-T and incubated with HRP-conjugated secondary antibody for 1 hour at RT. Antibody signals were detected by incubating membrane with SuperSignal™ West Dura Extended Duration Substrate (ThermoFisher). Images were acquired by Sapphire Biomolecular Imager (Azure biosystems) and processed using Adobe Photoshop CC 2018. Relative protein amount was measured by calculating the pixel intensity using ImageJ (National Institute of Health). Immunoblots presented in all figures are representatives of at least three independent experiments. Antibodies used in this study can be found in **Supplementary Table 2**.

### Cell Growth Inhibition Assay

Cells were seeded in opaque-walled 96-well microplates at appropriate densities according to the growth curves (data no shown). On the following day cells were dosed with compounds. After 7 days treatments, cells viability was measured by adding 30μL CellTiter-Glo reagent (Promega) and incubated for 10 min at RT. Luminescence was measured by Envision plate reader (Perkin Elmer). For synergy analysis, the survival rates of the cells upon different treatment combinations were calculated based on the luminescence and analyzed by Combenefit software. The bliss independence model was used to analyzed the interaction between two tested articles to determine if the interaction between those two tested articles was synergistic, independent or antagonistic.

### Cell Cycle Analysis

Cells were fixed with 70% ethanol and then washed with PBS for 3 times (5 min each), after the fixation, they were stained with 150 μL PI/RNase Staining Buffer incubated 15 min at RT in the dark. DNA content were determined using a FACS Canto II flow cytometer (BD Biosciences).

### Apoptosis Assay

For analysis of apoptosis, treated cells were stained with PE Annexin V Apoptosis Detection Kit I (BD) in accordance with the manufacturer’s instructions. Flow cytometry data were acquired using a FACS Canto II flow cytometer (BD Bioscience) and analyzed using FACS Diva software (BD Bioscience).

### γH2AX Analysis

Cells treated with compounds or cisplatin were harvested at indicated time points, fixed with IC Fix buffer overnight and permeabilized with True-Phos™ Perm Buffer (BioLegend) for 1 h at -20°C. After washed with PBS for 3 times (5 min each), cells were stained with anti-γH2AX (phospho S139) antibody for 1 h at 4°C. Cells were then washed and incubated with Alexa Fluor^®^ secondary antibody for 1 h at 4°C. The fluorescence was determined by flow cytometry (BD Biosciences) and analyzed using FACS Diva software (BD Biosciences).

### Flow Cytometric Analysis of Cell Surface PD-L1

To evaluate cell surface PD-L1 levels, cells were suspended in 100 μL of cell staining buffer and incubated with PE anti-human CD274 (PD-L1) antibody at 4°C for 30 min. Then cells were washed in PBS for 3 times (5 min each). Flow cytometry data were acquired using a FACS Canto II flow cytometer (BD Biosciences) and analyzed using FACS Diva software (BD Biosciences).

### *In Vivo* Studies

Daudi xenograft mouse model was established by subcutaneously implantation of 10^7^ cells into the female BABL/c nude mice at the age of 6-8 weeks. When tumors reached approximately 100-120 mm^3^, the mice were randomly grouped into five groups (n=6) and treated with vehicle control orally every day, 30 mg/kg or 100 mg/kg Cpd-4 orally every day, 2 mg/kg Cisplatin intraperitoneally every week, or combination of 30 mg/kg Cpd-4 and 2 mg/kg Cisplatin. Cpd-4 was formulated in in 30% PEG400 (Sigma) and 70% 10% vitamin E TPGS (Sigma) in water. Cisplatin was reconstituted in normal saline. Cpd-4 was administrated starting from Day 0 and Cisplatin was administrated starting from on Day1. Cisplatin was administrated 4 hours after the treatment of Cpd-4 at day 1, 8 and 15. Tumor volume was monitored twice a week.

The long diameter (*a*) and the short diameter (*b*) of the tumor were measured using caliper and the tumor volume (*v* was calculated using the following formula:


$$V=0.5\times a\times b^{2}$$

Tumor growth inhibition was calculated using the formula:


$$\text{TGI}(\%)=\frac{\left(1-({\text{V}}_{t}(\text{treatment group})-{\text{V}}_{0}(\text{treatment group})\right)}{{\text{V}}_{t}(\text{vehicle group})-{\text{V}}_{0}(\text{vehicle group}))}\times 100\%$$

V_0_ is the tumor volume of the animal when treatment starts; v_t_is the tumor volume of the animal someday after treatment. The statistics of tumor volume was analyzed by two-way ANOVA followed by Tukey’s multiple comparisons test using GraphPad Prism 8.0.

### T-Cell Killing Assay

MDA-MB-231 cells were seeded in 96-well plates. On the following day, cells were treated with DMSO or Cpd-4 for 48h. Human peripheral blood mononuclear cells (STEMCELL) were activated with 100 ng/mL CD3 antibody, 100 ng/mL CD28 antibody, and 10 ng/mL IL2 (BioLegend) and then cocultured with MDA-MB-231 cells at 10:1 ratio. The co-cultured cells were treated with or without PD-L1 antibody for 24h. Cell viability was measured by CellTiter-Glo reagent.

### Statistical Analysis

Unless stated otherwise ordinary one-way ANOVA followed by Dunnett’s multiple comparisons test using GraphPad Prism 8.0 was used to determine the significances of differences. *, P<0.05; **, P <0.01; ***, P <0.001; ****, P <0.0001. P-values of <0.05 were considered statistically significant and P-values of <0.1 were considered meaningful.

## Results

### High RAD51 Expression Is Associated With Poor Clinical Outcome

**Figure 1:**
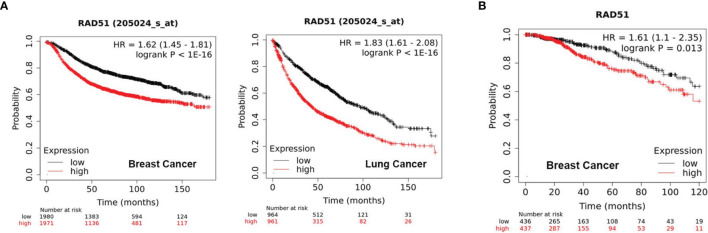
High RAD51 expression is associated with poor clinical outcome. **(A)** Kaplan-Meier Plots showing the probability of relapse-free survival (RFS) in breast cancer patients (n = 3951, left) and overall survival (OS) in lung cancer patients (n = 1925, right), who were stratified by the median of RAD51 gene expression. **(B)** Kaplan-Meier Plots showing the probability of OS in breast cancer patients (n = 873), who were stratified by the median of RAD51 protein expression. *P* value was calculated using the log-rank test.

Initially, we evaluated the significance of RAD51 as a potential therapeutic target by analyzing of public datasets with large sample sizes. The KM Plotter Online Tool (kmplot.com) (17) was used to associate RAD51 expression with clinical outcome for more than 1000 patients with breast or lung cancer. The analysis revealed that tumors with high RAD51 mRNA expression is significantly associated with poor outcome in both cancer types (**Figure 1A**). Further, high expression of RAD51 protein was also found to be significantly associated with shorter overall survival (OS) based on RPPA data retrieved from TCGA (**Figure 1B**). Overall, these data suggest that RAD51 overexpression is a negative prognostic marker and thus a promising therapeutic target.

### Discovery and Mechanism of Action of a Novel Class of RAD51 Inhibitors

**Figure 2:**
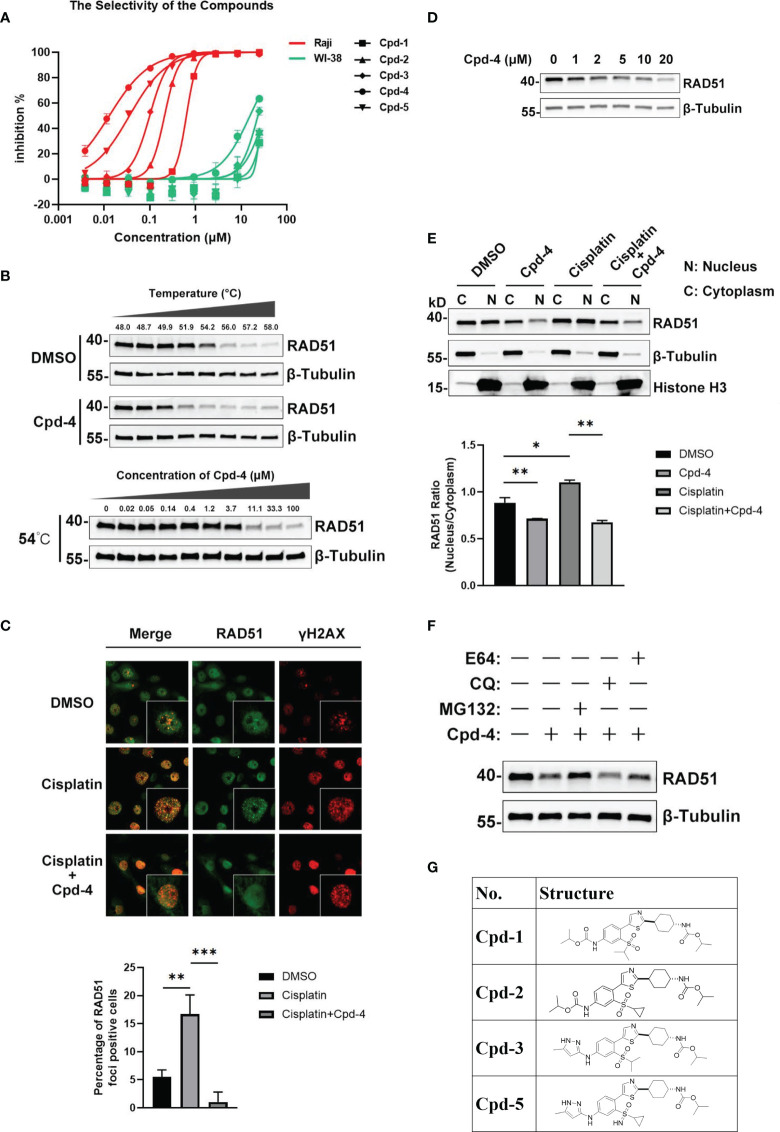
Discovery and mechanism of action of a novel class of RAD51 inhibitors. **(A)** Representative dose response curve of a series of RAD51 inhibitors on Raji and WI-38 cell line generated in GraphPad Prism. **(B)** Comparison of thermo stability of endogenous RAD51 protein with or without the treatment of compounds. β-Tubulin served as the negative control. In the top panel, Z138 cells were treated with 20 μM Cpd-4. **(C)** Image analysis of DNA damage and repair marker upon the compound treatment. HCC1937 cells were treated with DMSO (upper and middle panels) or Cpd-4 (10μM, lower panel) for 2 days, then cells were exposed to cisplatin (10μM) for 2 h (middle and lower panels) and stained 5 h later. Quantification shows fraction of cells with ≥10 RAD51 foci. **(D)** Western blot analysis of endogenous RAD51 protein level in HCC-1937 cells after the treatment of compounds for 24 h. **(E)** Subcellular fraction of RAD51 protein in HCC-1937 cells were separated by using the NE-PER Nuclear Cytoplasmic Extraction Reagent Kit (Pierce) and analyzed by western blot. β-Tubulin and histone H3 were used as the cytoplasmic and nuclear markers, respectively. **(F)** Compounds treatment induced RAD51 protein degradation can be blocked by proteasome inhibitor (MG132) but not lysosome inhibitor (CQ or E64). All data represent mean ± SD based on at least three biological repeats. **(G)** Structures of representative compounds. *, P<0.05; **, P <0.01; ***, P <0.001.

Up to now, there is only a limited number of useful inhibitors for RAD51. The widely used RAD51 inhibitor B02 inhibits DNA strand exchange activity (18, 19). However, it exhibits a relatively weak inhibitory potency regarding cell growth. We therefore aimed to identify a unique class of inhibitors with a novel molecular mechanism of action. To this end, we designed and synthesized about 100 diverse compounds in-house which were tested for their cellular potency and pharmacokinetics parameters to yield more drug-like inhibitors. Because Raji cells are characterized by genomic instability induced by c-MYC overexpression (16), this cell line was used for proliferation screening against normal WI-38 cells. This initial screening allowed the identification of five compounds, named as Cpd-1, Cpd-2, Cpd-3, Cpd-4, and Cpd-5, exhibiting potent antiproliferative effects in Raji cells with nanomolar IC_50_ values which did not affect normal cell viability (IC_50_ > 10 μM) (**Figure 2A**). Since these compounds displayed similar cellular potency, they were used interchangeably in subsequent studies.

To investigate the mechanism of action of these novel RAD51 inhibitors, we performed different molecular assays. We first determined intracellular target engagement using the commonly used cellular thermal shift assay (CETSA) (20, 21). Here the RAD51 inhibitors induced a considerable destabilization of cellular RAD51 upon increased temperatures when compared to DMSO (**Figure 2B**). Furthermore, a concentration-dependent destabilization of RAD51 induced by compounds at 54°C was observed (**Figure 2B**). These results suggest a specific binding of the compound to RAD51 in cells and, thus, implicating RAD51 as a *bona fide* target of the newly developed compounds.

For HR-mediated DNA damage repair, RAD51 foci formation is a critical step and unrepaired DNA damage results in reduced cellular survival. To determine if the antiproliferative effect of identified small molecules was mediated *via* inhibition of RAD51 foci, we investigated the effect of RAD51 inhibitors on RAD51 foci formation and the DNA damage marker γH2AX following treatment with a DNA damaging agent. To this end, BRCA1 mutant HCC-1937 breast cancer cells were treated with either DMSO or the RAD51 inhibitor for 2 days before cisplatin treatment. The results demonstrated detectable levels of RAD51 foci in cultured cells without cisplatin (**Figure 2C**). As expected, exposure to cisplatin increased RAD51 foci formation (**Figure 2C**) in cells while treatment with the RAD51 inhibitor inhibited respective cisplatin-induced RAD51 foci formation (**Figure 2C**). Consistently the combination of RAD51 inhibitors with cisplatin led to an increase of γH2AX, indicative of an accumulation of DNA damage (**Figure 2C**). This result suggests that the inhibitor directly impaired the formation of RAD51 foci. We also performed a similar experiment using BRCA1 wildtype MDA-MB-468 breast cells. Comparing to BRCA1 mutant HCC-1937 cells, though cisplatin treatment induced less RAD51 foci formation and γH2AX increase, the RAD51 foci formation can also be inhibited upon RAD51 inhibitor treatment (**Figure S1**).

To investigate the mechanism of the attenuated RAD51 foci formation by RAD51 inhibition, we evaluated the effects of RAD51 inhibitors on RAD51 protein levels and its subcellular distribution. Western blot analysis revealed that the inhibitor treatment resulted in a concentration-dependent decrease in RAD51 protein levels in HCC-1937 cells (**Figure 2D**). We also determined that the nuclear localization of RAD51 was reduced after RAD51 inhibitor treatment both in the presence and the absence of cisplatin (**Figure 2E**). These findings suggest that the RAD51 inhibitor prevented the RAD51 foci formation, at least in part, by reducing nuclear accumulation and stability of RAD51. Furthermore, we found that RAD51 protein degradation induced by the RAD51 inhibitor can only be prevented when cells were treated with the proteasome inhibitor MG132, but not the lysosome inhibitor chloroquine (CQ) or E64 (**Figure 2F**), suggesting that the RAD51 inhibitor facilitates proteasomal degradation of RAD51 protein.

To investigate how these new compounds function differently from the RAD51 inhibitors currently in use, we performed docking simulations between RAD51 inhibitors and RAD51 protein. The binding mode of one representative RAD51 inhibitor Cpd-5 to RAD51 protein, as obtained by docking simulations, displays some points of interaction similar to those of the crystallographic BRC4-RAD51 complex (**Figure S2A**). Specifically, the docking model suggests that (i) the cyclopropane in a hydrophobic pocket outlined by the side chains of Tyr202, Ala203, Arg204, Leu214, Ala218 of RAD51; (ii) the NH group of the pyrazole-3-amine group forms hydrogen bonds interaction with the Gln217 of RAD51; the NH group of the methylcarbamate forms hydrogen bonds interaction with the Ala201 of RAD51. In addition, the model suggests that the pyrazole ring is likely to form the Π-Π interaction with the H210. By analyzing the electrostatic potential of RAD51 pockets and Cpd-5, we found that molecules can better form electrostatic complementarity with RAD51 pockets (**Figure S2B**). The chemical structures of our RAD51 inhibitors are illustrated (**Figure 2G**).

### RAD51 Inhibition Attenuates Growth of Various Types of Tumor Cells and Induces Cell Cycle Arrest

**Figure 3:**
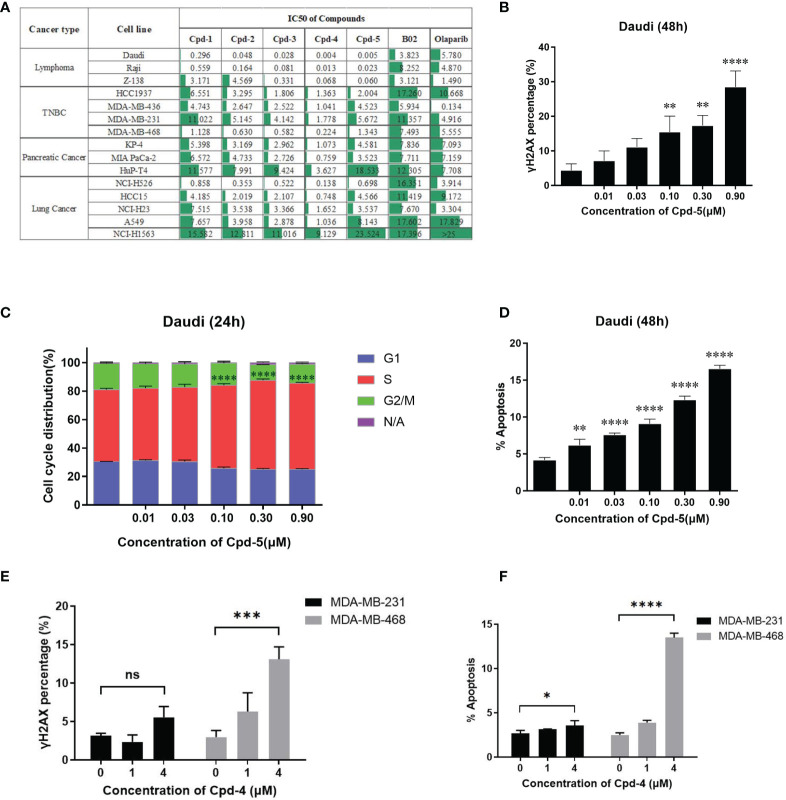
RAD51 inhibition attenuates growth of various types of tumor cells and induces cell cycle arrest. **(A)** Estimated IC_50_ values (μM) of a series of RAD51 inhibitors and Olaparib on different cancer cell lines based on the four‐parameter dose response curve generated in GraphPad prism. Data are representative of at least three independent experiments. **(B)** Dose-dependent γH2AX expression under compound treatment was analyzed by flow cytometry. Statistical analysis was performed comparing Cpd-5 treated cells with non-treated cells. **(C)** Cell cycle analysis of Daudi cells after compound treatment for 24 hours. Cells were stained with PI and images were analyzed by flow cytometry. N/A (non-assigned) represents cell populations where signal intensities exceeded the threshold to accurately determine the cell cycle phase. Statistical analysis was performed using percentage of cells in S-phase treated with Cpd-5 comparing with non-treated cells. **(D)** Induction of apoptosis in Daudi cells by compound treatment in a dose-dependent manner at 48 h. Statistical analysis was performed comparing Cpd-5 treated cells with non-treated cells. **(E)** Quantitative data from flow cytometry analysis showing percentage of γH2AX positive cells in MDA-MB-468 and MDA-MB-231 cell lines after compound treatment. **(F)** Analysis of apoptosis by flow cytometry in MDA-MB-468 and MDA-MB-231 cell lines following treatment with compounds for 48 h. All data presented with mean ± SD are based on at least three biological repeats. *, P<0.05; **, P <0.01; ***, P <0.001; ****, P <0.0001; NS, not significant.

Next, we examined the effects of the five identified compounds on the proliferation of 15 human tumor cell lines from four cancer types (**Figure 3A**). The most significant inhibition of cell growth was observed for lymphoma cell lines. Of the compounds tested, Daudi showed the most response to Cpd-5 (IC_50_ = 5 nM) and Cpd-4 (IC_50_ = 4 nM). Notably, compared with B02, this novel class of inhibitors can decrease the IC_50_ values by more than 100-fold in some of the cell lines. These data suggest that the newly identified RAD51 inhibitors have a broad antiproliferative activity against various types of cancer cells. Because PARP inhibitors are reported to exhibit increased sensitivity in cells with HR deficiency (22), we were interested to know the potential of olaparib sensitivity as a biomarker of response to RAD51 inhibitors. The 15 cell lines exhibited diverse sensitivity to olaparib, with IC_50_ values ranging from 0.134 μM to over 25 μM. However, we did not observe any significant correlation between sensitivity of cells to olaparib and RAD51 inhibitors, suggesting that response to olaparib does not appear to be a predictive biomarker for RAD51 inhibitor sensitivity in cancer cells. To evaluate the effects of RAD51 inhibitors on inducing DNA damage, we analyzed γH2AX expression and found a dose response for γ-H2AX following exposure to the RAD51 inhibitor Cpd-5 with statistically significant increase of γ-H2AX expression at 0.1 μM and above (*P*<0.01, **Figure 3B**). We further analyzed the effects of the RAD51 inhibitor on cell cycle progression and apoptosis by flow cytometry. This analysis showed that RAD51 inhibition caused a dose-dependent S-phase arrest of the cell cycle, consistent with its central role in HR, which occurs preferentially during S-phase. Similarly, statistically significant increase of cells in S-phase has been observed when Cpd-5 concentration was 0.1 μM and above (*P*<0.0001, **Figure 3C**). Additionally, Daudi cells exposed to escalating doses of the RAD51 inhibitor displayed a dose-dependent statistically significant increase in apoptosis (*P*<0.01, **Figure 3D**). Together, these data suggest that these next generation RAD51 inhibitors impaired cell growth through cell cycle arrest in S-phase and elevated apoptosis.

To further confirm that the difference in sensitivity of cells to the compound is due to the mechanism of action instead of off-target toxicity, two TNBC cell lines (MDA-MB-231 and MDA-MB-468) with a 10-fold difference in IC_50_ were selected for a comparison of DNA damage and apoptosis. At the same concentration of compound, the increase in γH2AX expression and apoptosis in MDA-MB-468 cells was greater than that in MDA-MB-231 cells (**Figures 3E, F**), accounting for the higher sensitivity of MDA-MB-468 cells to the RAD51 inhibitor.

### RAD51 Inhibition Enhances the Anti-Tumor Effect of Chemotherapy Agents

**Figure 4:**
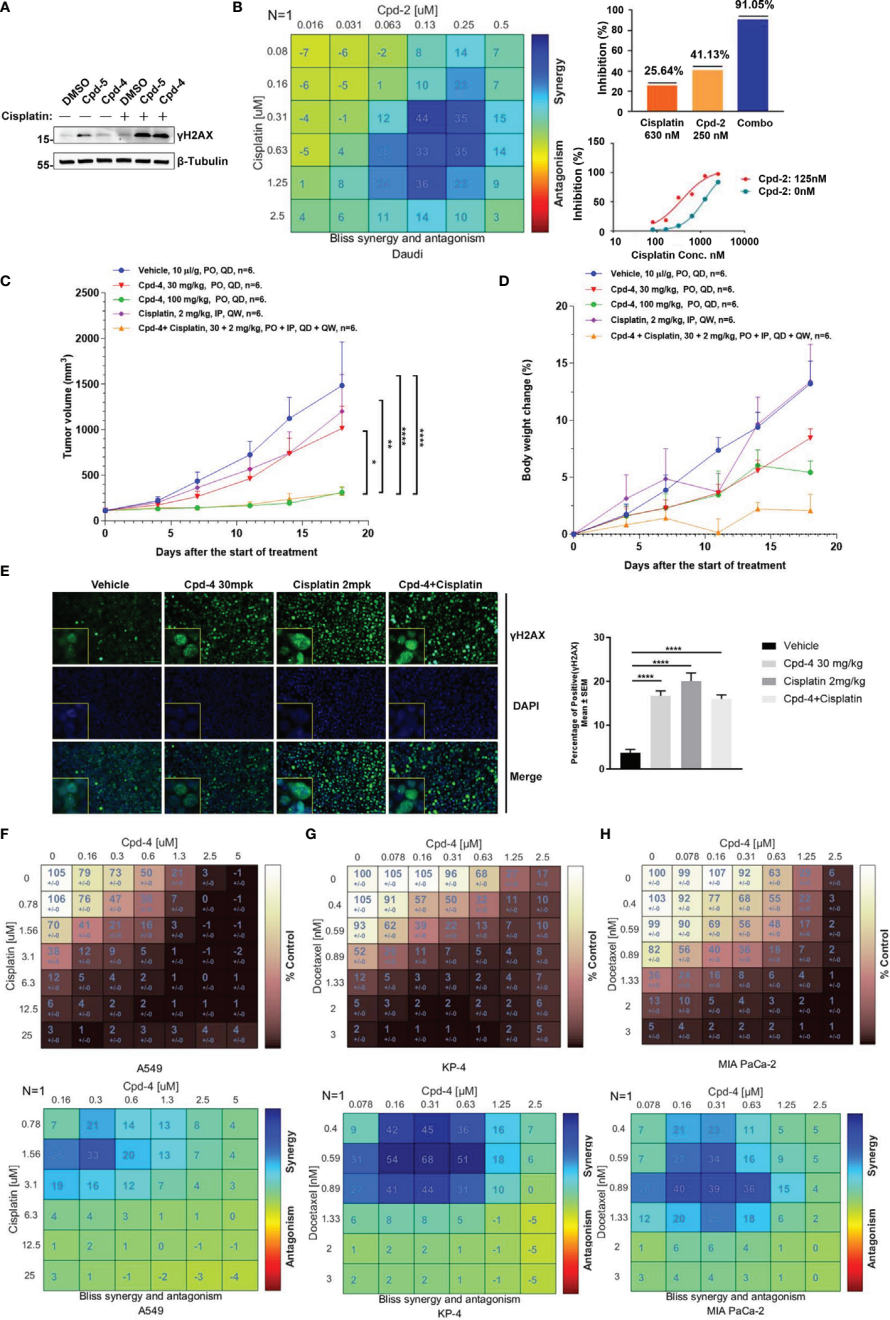
RAD51 inhibition enhances the anti-tumor effect of chemotherapy agents. **(A)** Western blot analysis of γH2AX expression in cell lysate. Daudi cells were co-cultured with DMSO, Cpd-5 (50 nM) and Cpd-4 (25 nM) for 72 h, then cells were treated with 30 μM cisplatin for 2 h and recovered for 5 h. β-Tubulin served as loading control. **(B)** Synergy effect of compounds and cisplatin in Daudi cells. Cells were dosed with Cpd-2 and cisplatin in a 6×6 concentration grid in 96-well plate for 7 days. Cell viability was determined with CellTiter-Glo reagent. The experimental data were analyzed with bliss synergy model using the Combenefit software (left panel) and GraphPad Prism software (right panel). Data are representative of at least two independent experiments. **(C, D)** The anti-tumor effects of Cpd-4 and cisplatin in the Daudi xenograft mouse model. The Daudi tumor bearing female BALB/c nude mice were administrated with Cpd-4, cisplatin or combination and the tumor volume and the body weight change are graphed in **(C, D)**, respectively (N=6 for each group). The error bars represent Standard Error of Mean. PO, oral garage; QD, once daily; IP, intraperitoneal injection; QW, once a week. P value was calculated based on the tumor volume using two-way ANOVA. *, P<0.05; **, P <0.01; ****, P <0.0001. **(E)** γH2AX expression in tumor tissue analyzed by fluorescence histochemistry. For quantification, nuclei with ≥5 foci were counted as γH2AX-positive cells. A total of 4 field with >100 cells were counted in each mouse. Mean ± SEM. Scale bar, 100 μM. **(F)** Synergy analysis of compounds and cisplatin in A549 cell lines. Upper panel, cell viability. Lower panel, bliss synergy score. **(G, H)** Synergy effect of compounds and docetaxel in MIA PaCa-2 and KP-4 cell lines. Data are representative of at least two independent experiments.

Given the crucial role for RAD51 in repairing DSBs induced by chemotherapy, we explored whether RAD51 inhibition could sensitize cancer cells to chemotherapeutic drugs. To this end, we detected γH2AX levels in cells by western blot as a measure of DNA damage. Daudi cells were incubated with RAD51 inhibitors for 72 h before a 2 h co-incubation with cisplatin or DMSO. We observed that the RAD51 inhibitors greatly potentiated cisplatin-induced DSBs, evidenced by the dramatic increase of γH2AX levels in cells treated with cisplatin in combination with RAD51 inhibitors compared with those treated only with cisplatin or RAD51 inhibitors (**Figure 4A**). Accordingly, we observed significantly improved cytotoxicity of the RAD51 inhibitor in combination with cisplatin as demonstrated by a 3.4-fold shift in IC_50_ (**Figure 4B**). Synergy analysis employing the Bliss model (Combenefit) revealed a strong synergy for the combination of RAD51 inhibitors and cisplatin (**Figure 4B**). Specifically, limited doses of RAD51 inhibitor (0.25 μM) and of cisplatin (0.63 μM) exerted only mild effects on cell viability on their own, whereas the combination of RAD51 inhibitor and cisplatin inhibited cell viability by 90%, demonstrating a strong synergistic effect (**Figure 4B**).

To further evaluate the synergistic effect of RAD51 inhibitors with cisplatin *in vivo*, BALB/c nude mice bearing Daudi tumors were treated with RAD51 inhibitor and/or cisplatin. The RAD51 inhibitor Cpd-4 alone showed dose-dependent anti-tumor efficacy, with TGIs of 34.3% and 85.6% at 30 mg/kg and 100 mg/kg, respectively. Cisplatin alone at 2 mg/kg did not show any significantly anti-tumor activity with TGI of 20.7%. Low dose combination treatment of Cpd-4 (30 mg/kg) and cisplatin (2 mg/kg) presented significantly better anti-tumor efficacy than either monotherapy with TGI of 86.2%, indicating a synergistic effect with acceptable tolerability (**Figures 4C, D**). Subsequent immunofluorescence histochemistry carried out on Daudi xenograft tissue indicated increased γH2AX expression in groups treated with Cpd-4, cisplatin or drug combination (**Figure 4E**) compared to vehicle group.

In addition to lymphoma, we also verified the synergistic effect of RAD51 inhibitors with cisplatin on solid tumor cells. Our results showed a strong combination effect of RAD51 inhibitors and cisplatin in A549 lung cancer cells (**Figure 4F**). Furthermore, we also investigated RAD51 inhibition in context of pancreatic cancer cell. In this tumor type, the microtubule-targeting agent Docetaxel combined with other DNA-damaging agents represents a first-line chemotherapeutic regimen. Although the underlying action remains largely unknown, it is proposed that microtubule-targeting agents cause cytoplasmic retention of DNA repair proteins, and thus enhance DNA damage (23). We postulated that inhibition of RAD51, a protein essential for DNA repair, would increase Docetaxel sensitivity. To test this hypothesis, we used two representative pancreatic cancer cell lines (KP-4 and MIA PaCa-2) and analyzed the effect of synergy. We observed that the combination of RAD51 inhibitors and Docetaxel displayed a strong synergy in both cell lines (**Figures 4G, H**). Collectively, these data demonstrate the potential clinical utility for RAD51 inhibitors in combination with chemotherapy.

### RAD51 Inhibition Synergizes With DDR-Targeting Agents on Cell Proliferation

**Figure 5:**
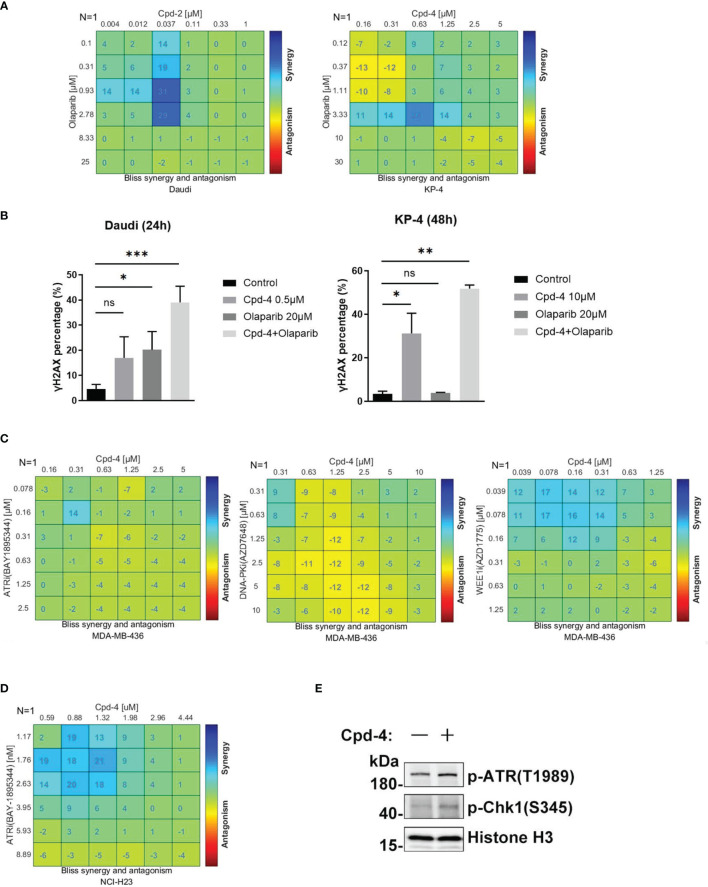
RAD51 inhibition synergizes with DDR-targeting agents on cell proliferation. **(A)** Synergy effect of compounds and olaparib in Daudi and KP-4 cells. Cells were dosed with Cpd-4 and Olaparib in a 6×6 concentration grid in 96-well plate for 5-7 days. Cell viability was determined with CellTiter-Glo reagent. The experimental data were analyzed with bliss synergy model using the Combenefit software. Data are representative of at least two independent experiments. **(B)** γH2AX expression analyzed after cells exposed to drugs combination. Cells were treated with DMSO, Cpd-4, Olaparib or drug combination for 24 h (Daudi) or 48 h (KP-4), then cells were stained with γH2AX antibody and analyzed by flow cytometry. **(C)** Synergy analysis of compound and ATRi (BAY1895344), DNA-PKi (AZD7648) or WEE1i (AZD1775) combination in MDA-MB-436 cells. **(D)** Synergy analysis of compound and ATRi (BAY1895344) combination in NCI-H23 cell lines. **(E)** ATR-Chk1 signaling was up-regulated under the compound treatment in NCI-H23 cells. Cells were treated with DMSO or 10 μM Cpd-4 for 6 h before subjected to western blot analysis. *, P<0.05; **, P <0.01; ***, P <0.001, ns, not significant.

PARP family proteins are activated upon binding to damaged DNA and have crucial roles in detecting SSB, recruiting DDR machinery and stabilizing replication forks during repair (24). PARP inhibitors are approved therapies for a number of cancers including breast cancers, pancreatic adenocarcinoma and ovarian cancer that carry HR-related mutations based on the concept of synthetic lethality (25). Given the role of RAD51 in HR-mediated DNA damage repair, we further explored the synergistic effect of RAD51 inhibition with olaparib in Daudi and KP-4 cells. As shown in **Figure 5A**, a moderate synergy was observed in both cell lines. These synergy effects were further confirmed by γH2AX expression analysis (**Figure 5B**). Next, we explored the possibility of the combination of RAD51 inhibitor and other DDR-targeting agents. While the RAD51 inhibitors synergized with WEE1 inhibition, no apparent synergistic effect was observed when combined with ATRi or DNA-PKi in MDA-MB-436 breast cancer cells (**Figure 5C**). Previous studies reported that ATM was upregulated when ATR signaling was blocked (26), implying ATM compensation for ATR deficiency. Therefore, we investigated the synergy between RAD51 inhibition and ATRi in ATM-null cells. In the ATM-deficient NCI-H23 cells, the RAD51 inhibitor showed profound synergy with ATRi with regard to inhibition of cell proliferation (**Figure 5D**). Consistent with this, we found an activation of ATR signaling when RAD51 was inhibited in NCI-H23 cells (**Figure 5E**).

### RAD51 Inhibition Induces PD-L1 Upregulation and Shows Potential for Combination With PD-L1 Immune Checkpoint Blockade

**Figure 6:**
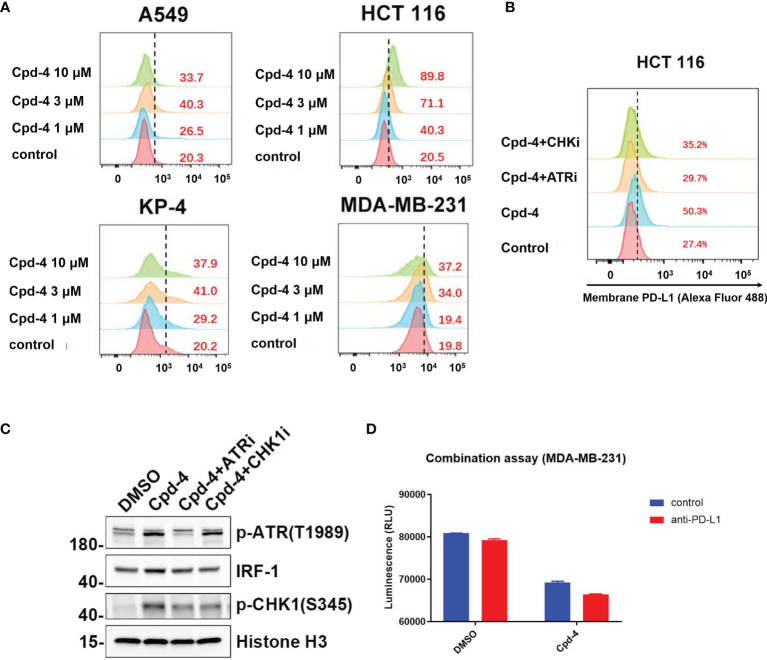
RAD51 inhibition induces PD-L1 upregulation and shows potential for combination with PD-L1 immune checkpoint blockade. **(A)** RAD51 inhibitor upregulates PD-L1 expression in various cell lines. MDA-MB-231, A549, KP-4 and HCT 116 cells were treated with indicated concentration of Cpd-4 for 24 h, and cell surface PD-L1 was analyzed by flow cytometry. **(B)** RAD51 inhibition induced PD-L1 expression depends on the activity of ATR-Chk1 signaling. HCT 116 cells were treated with ATRi (AZD6738, 10 μM) or Chk1i (MK8776, 1 μM) inhibitor 1 h prior to RAD51 inhibitor (Cpd-4, 2 μM) treatment. Cell surface PD-L1 expression was examined after 24 hours. **(C)** ATR-Chk1 signaling promotes IRF-1 expression after RAD51 inhibition in HCT 116 cells. Cells were treated with DMSO, ATRi or CHKi for 2 hours and then treated with DMSO or Cpd-4 for 4 h. The levels of p-ATR, IRF1 and p-Chk1 were examined by western blot after indicated compound treatment. **(D)** Quantitation showing cell viability following treatment with Cpd-4 (10 μM), PD-L1 antibody (PD-L1 Ab; 10 mg/mL), or the combination cocultured with activated PBMCs for 72 hours. Cell viability were determined with CellTiter-Glo reagent.

Double strand break (DSB) repair is sufficient to induce PD-L1 expression in cancer cells through ATR/Chk1 signaling axis (27). Because RAD51 inhibition induces DSBs as evidenced by an accumulation of γH2AX foci, we wanted to determine whether PD-L1 expression would be increased. The FACS analysis revealed that RAD51 inhibitor treatment increased PD-L1 expression in TNBC (MDA-MB-231), lung cancer (A549), pancreatic cancer (KP-4) and colon cancer (HCT 116) cell lines in a dose-dependent manner (**Figure 6A**).

Next, we analyzed whether RAD51 inhibition induced PD-L1 expression is mediated by ATR/Chk1/IRF-1 signaling. HCT 116 cells exposed to the RAD51 inhibitor displayed a 5-fold increase of PD-L1 expression compared with DMSO-treated cells (**Figure 6B**). Strikingly, the induction of PD-L1 expression upon RAD51 inhibition was significantly suppressed by a specific inhibitor of ATR or Chk1, suggesting that PD-L1 upregulation requires ATR-Chk1 signaling after RAD51 inhibition. To confirm the ATR activation, we measured ATR phosphorylation at Thr-1989 and Chk1 phosphorylation at Ser-345. RAD51 inhibitor treatment led to a dramatic increase in p-ATR and p-Chk1 levels (**Figure 6C**), consistent with what has been observed in NCI-H23 cells (**Figure 5E**). Previous studies reported that IRF-1 is a potential effector downstream of ATR-Chk1 signaling and modulates PD-L1 expression (27, 28). As expected, we found that the RAD51 inhibitor exposure resulted in an increase in levels of IRF-1, which could be suppressed by ATR or Chk1 inhibitor treatment (**Figure 6C**). Collectively, these findings show that RAD51 inhibition-mediated IRF1 upregulation, a downstream component of ATR/Chk1 signaling, is a critical mechanism underlying PD-L1 expression regulation in cancer cells.

RAD51 inhibition induced PD-L1 upregulation may result in increased binding of PD-1 and affect T-cell functions. We therefore tested T-cell killing in context of the combination of PD-L1 antibody and RAD51 inhibitor. The results showed that the combination of RAD51 inhibition and PD-L1 blockade was more effective than each agent alone in inducing T-cell killing (**Figure 6D**), suggesting potential for combination treatment of RAD51 inhibitors with PD-L1 immune checkpoint blockade.

## Discussion

Due to the prominent role of RAD51 to maintain the genomic stability, we set out to identify a small molecule that would inhibit its biological function as a strategy for cancer therapy. To accomplish this goal, we have screened about 100 diverse compounds synthesized in-house using an *in vitro* cell proliferation assay. Here we describe what is to our knowledge the first small molecule inhibitor of RAD51 with low-nanomolar IC_50_ values regarding antiproliferative effects *in vitro.* The RAD51 inhibitor Cpd-4, as a single agent, demonstrated dose-dependent antitumor activity in a xenograft model. Corresponding increased levels of γH2AX in the excised tumors suggest that the antitumor effects were a direct consequence of HR disruption. Using CETSA, we found that the RAD51 protein got thermally destabilized upon addition of Cpd-4 inside cells, further supporting a specific RAD51 inhibitory activity of this compound. Mechanism of action analysis indicated that the novel inhibitor prevented RAD51 foci formation by altering the nucleocytoplasmic distribution and by acceleration of RAD51 degradation. Unlike this novel class of inhibitors, the older-generation RAD51 inhibitors have a different mechanism of action (29). For instance, B02 inhibits DNA strand exchange activity of RAD51, while IBR2 disrupts RAD51 oligomerization through inhibition of the BRC motif-RAD51 interaction (15). Regardless of the different mechanism of action, micromolar potencies of these compounds in human cells present significant obstacles for their potential clinical utility.

Our work also reveals a broad antiproliferative response of cancer cells originating from various organs by RAD51 inhibition. Hence, our identified inhibitors could be expected to be efficacious in various solid tumors. However, we show that various solid tumor cell lines differentially responded to RAD51 inhibition, with MDA-MB-468 and NCI-H526 being sensitive whereas MDA-MB-231 being less sensitive to RAD51 inhibition. This could be due to differences of their genetic backgrounds, among other factors. Indeed, increasing evidence illustrates that most synthetic lethal effects appear to be highly context-dependent, in other words the effects are only observed in one specific genetic background (30). As such, in PDAC cell lines, deletion of ATM was found to sensitize the cells to ATR inhibition (31). Future work will need to aim at overcoming the current challenge to identify which genetic backgrounds confer sensitivity to the RAD51 inhibition. To this end, further assessment of the efficacy of the inhibitor using solid human tumor xenografts is warranted.

Previous reports have demonstrated the abilities of the inhibitors targeting DDR proteins such as DNA-PK and ATR to sensitize cells to chemotherapeutic agents. For example, the selective DNA-PK inhibitor, AZD7648, enhanced doxorubicin efficacy in both xenograft and patient-derived xenograft (PDX) models (32). In addition, several ATR inhibitors in combination with chemotherapy reveal preclinical activities and have been advanced to clinical trials (34). This is consistent with a role for DDR kinases in repairing cytotoxic DNA damages. In agreement with this, our study showed that inhibition of RAD51 is synthetic lethal with cisplatin in Daudi cells and with Docetaxel in pancreatic cells. This synergistic effect was associated with DNA damage accumulation, as evidenced by elevated levels of γH2AX in respective cells (**Figure 4A**). Moreover, our data provide direct evidence that Cpd-4 and cisplatin combination enhanced antitumor efficacy *in vivo*, suggesting that a combination treatment could be beneficial in patients.

In order to make PARP inhibitors more efficacious, efforts are continuously ongoing to develop targets of DDR pathway that can be proposed for an enhancement of the cancer response through synthetic lethality. As reported previously, patients with HR-deficient tumors demonstrated therapeutic benefits to PARP inhibitors (35). Given the critical role of RAD51 in commencing HR in cases of DSBs, we hypothesize that RAD51 inhibition could create an HR-deficient phenotype that likely synergizes with PARP inhibitors. Consistent with this hypothesis, we demonstrate that RAD51 inhibitors combined with olaparib increased accumulation of DNA damage, producing a synergistic effect on cancer cell growth inhibition. In support of our results, inhibitors of ATR and DNK-PK kinases, the key players of the DDR, were shown to sensitize cancer cells to olaparib (32, 33). However, only a modest synergy between RAD51 inhibition and olaparib was observed, in contrast to a strong synergy with chemotherapy. Presumably, this might be due to lower base line levels of endogenous DSBs in the absence of DNA damage induction or due to bypass within the DDR machinery.

We further examined synergy of RAD51 inhibition with a number of inhibitors against DDR kinases that are currently under clinical evaluation. Our data showed that a weak synergy was observed with each inhibitor in the MDA-MB-436 cell line with the exception of the WEE1 inhibitor. The WEE1 inhibitor AZD1775 inhibits CDK1 phosphorylation, resulting in premature mitotic entry and cell death. The synergistic interaction between RAD51 and WEE1 inhibition probably reflects the importance of targeting both the cell cycle checkpoints and DSB repair pathways simultaneously. Another interesting observation is that the combination between RAD51 and ATR inhibition were more effective in the ATM-deficient H23 cell line model but, in contrast, little synergy in MDA-MB-436 cells with functional ATM. Our findings show that the RAD51 inhibitor increased ATR activation in H23 cells, as judged by phosphorylation of ATR and its downstream target Chk1, which is in line with another study suggesting that RAD51 inactivation increased sensitivity to ATR and Chk1 inhibition (36). Previously, ATR inhibitor was shown to induce ATM activation as a compensatory response (37). This provides an explanation for the little synergy observed in MDA-MB-436 cells with functional ATM. Further exploration of other genetic vulnerabilities that sensitize to the combination are warranted to extend therapeutic options.

High expression of PD-L1 on tumor cells is well known to suppress antitumor T-cell responses and correlate with clinical responses to PD-1 therapy in cancer patients. Regulation of PD-L1 expression by small molecule inhibitors has been demonstrated to alter the efficacy of PD-L1/PD-1 immunotherapy in mouse models. For example, HDAC3 inhibitors synergize with PD-L1 blockade to enhance tumor regression by transcriptionally upregulating PD-L1 expression (38). Furthermore, genomic instability involved in PD-L1 regulation has been reported. DSBs induced by ionizing radiation or treatment with DNA damaging agents has recently been shown to lead to an increase of PD-L1 expression in cancer cells *via* an ATM/ATR/Chk1-dependent mechanism (27). Consistent with this report, we found that RAD51 inhibitors can increase surface PD-L1 levels in various tumor cells by activation of the ATR/Chk1 signaling and its downstream effector IRF1. A previous report suggested that IRF1 induction by STAT1/3 phosphorylation and its subsequent recruitment to the PD-L1 promoter by interferon gamma exposure is responsible for PD-L1 regulation (39), suggesting that the STATs-IRF1 pathway underlies the transcriptional upregulation of PD-L1. Such details should be further investigated to elucidate the mechanism by which DSBs trigger an IRF1 response to activate PD-L1 expression. Collectively, our findings reveal that RAD51 inhibition, leading to increased DNA DSBs, may be a rational strategy to be implemented in combination with PD-1 therapy to improve therapeutic outcome. Consistent with this notion, anti-PD-1 immune checkpoint blockade has recently been approved for the treatment of patients with microsatellite instability-high (MSI-H) or mismatch repair deficient (dMMR) colorectal cancer.

Overall, our findings establish that RAD51 inhibition could be used as a new prospect for cancer treatment with the potential to enhance the therapeutic window of many established therapeutic strategies across multiple cancer indications. We are optimizing those compounds to obtain a pre-clinical candidate RAD51 inhibitor which will be tested in future clinical studies.

## Data Availability Statement

The raw data supporting the conclusions of this article will be made available by the authors, without undue reservation.

## Ethics Statement

The animal study was reviewed and approved by Institutional Animal Care and Use Committee of the WuXi AppTec.

## Author Contributions

PG and LX made equal contributions to this work. They conceived and coordinated the study, designed the experiments, carried out data analysis, and wrote the paper. WL, ZJ, AL, TL, LL and XC designed and performed the experiments. CZ, MD, WY, and RT coordinated the study and made scientific contributions. RT conceived and coordinated the study, designed the experiments, carried out the data analysis and data interpretation, and revised the paper. All authors reviewed the results and approved the final version of the manuscript.

## Conflict of Interest

Authors PG, LX, CZ, WL, ZJ, AL, TL, LL, MD, WY, and RT were employed by Jiangsu Simcere Pharmaceutical Co., Ltd.

The remaining author declares that the research was conducted in the absence of any commercial or financial relationships that could be construed as a potential conflict of interest.

## Publisher’s Note

All claims expressed in this article are solely those of the authors and do not necessarily represent those of their affiliated organizations, or those of the publisher, the editors and the reviewers. Any product that may be evaluated in this article, or claim that may be made by its manufacturer, is not guaranteed or endorsed by the publisher.
