# Supplementary material for: Targeting the Homologous Recombination Pathway in Cancer With a Novel Class of RAD51 Inhibitors
Source: Front Oncol. 2022 May 13;12:885186. doi: 10.3389/fonc.2022.885186 (PMC9136011; doi:10.3389/fonc.2022.885186)
Supplement: Supplementary file 1 [file DataSheet_1.docx]

**Supplementary Materials for**

Targeting the homologous recombination pathway in cancer with a novel class of RAD51 inhibitors

Peng Gu^1^†, Liting Xue^1^†, Chunyan Zhao^1^, Wenjing Li^1^, Zhen Jiang^1^, Aiguo Liu^1^, Tingting Li^1^, Lu Liu^1^, Markus Decker^1^, Jinwen Shan^1^, Xiaoxuan Cheng^2^, Wenqing Yang^1^ & Renhong Tang^1^*

^1^State Key Laboratory of Translational Medicine and Innovative Drug Development, Jiangsu Simcere Pharmaceutical Co., Ltd., Nanjing, China

^2^Hangzhou foreign languages school, Hangzhou, China

†These authors have contributed equally to this work and share first authorship.

*** Correspondence:**Renhong Tang
renhong.tang@simceregroup.com

Keywords: RAD51, Small molecule inhibitor, DNA damage response, Homologous recombination, Synthetic lethality.

**Table S1. Cell lines and culture medium.**

| **Cell line** | **Culture medium** |
| --- | --- |
| Z-138 | IMEM +10% Horse Serum |
| Daudi | RPMI 1640 +10% FBS |
| Raji | RPMI 1640 +10% FBS |
| MDA-MB-468 | RPMI 1640 +10% FBS |
| MDA-MB-231 | RPMI 1640 +10% FBS |
| HCC-1937 | RPMI 1640 +10% FBS |
| MDA-MB-436 | DMEM +10% FBS |
| HuP-T4 | EMEM +10% FBS |
| KP-4 | RPMI 1640 +10% FBS |
| SKOV3 | McCoy's 5A + 10% FBS |
| NCI-H1563 | RPMI 1640 +10% FBS |
| HCC15 | RPMI 1640 +10% FBS |
| A549 | DMEM +10% FBS |
| H526 | RPMI 1640 +10% FBS |
| MIA PaCa-2 | DMEM +10% FBS + 2.5% Horse Serum |
| THP1 | RPMI 1640 +10% FBS + 0.05mM β-Mercaptoethanol |
| U937 | RPMI 1640 +10% FBS |
| NCI-H23 | RPMI 1640 +10% FBS |
| HCT 116 | McCoy's 5A + 10% FBS |

**Table S2. Antibody information and application.**

| **Antibody** | **Brand** | **Catalogue** | **Species** | **Application** | **Dilution** |
| --- | --- | --- | --- | --- | --- |
| Anti-RAD51 antibody | Abcam | ab63801 | Rabbit | IF, WB | 1:500, 1:1000 |
| Anti-γH2AX antibody | Millipore | 05-636 | Mouse | IF, IHC, WB | 1:500, 1:1000 |
| Anti-γH2AX antibody | Abcam | ab81299 | Rabbit | IHC | 1:200 |
| Anti-β-Tubulin antibody | Abcam | ab6046 | Rabbit | WB | 1:5000 |
| Anti-p-ATR(T1989) antibody | Abcam | ab227851 | Rabbit | WB | 1:1000 |
| p-Chk1(S345) | Abcam | ab58567 | Rabbit | WB | 1:1000 |
| IRF-1 | Abcam | ab186384 | Rabbit | WB | 1:5000 |
| PE anti-human PD-L1 Antibody | BioLegend | 393608 | Mouse | FC | 1:20 |
| Histone H3 | Cell Signaling | 4499S | Rabbit | WB | 1:5000 |
| Anti-Rabbit IgG H&L (Alexa Fluor® 488) | Abcam | ab150077 | Goat | IF | 1:1000 |
| Anti-Mouse IgG H&L (Alexa Fluor® 594) | Abcam | ab150116 | Goat | IF | 1:1000 |
| anti-Mouse IgG (H+L) Secondary Antibody, HRP | ThermoFisher | 31430 | Goat | WB | 1:5000 |
| anti-Rabbit IgG (H+L) Secondary Antibody, HRP | ThermoFisher | 31460 | Goat | WB | 1:5000 |

**
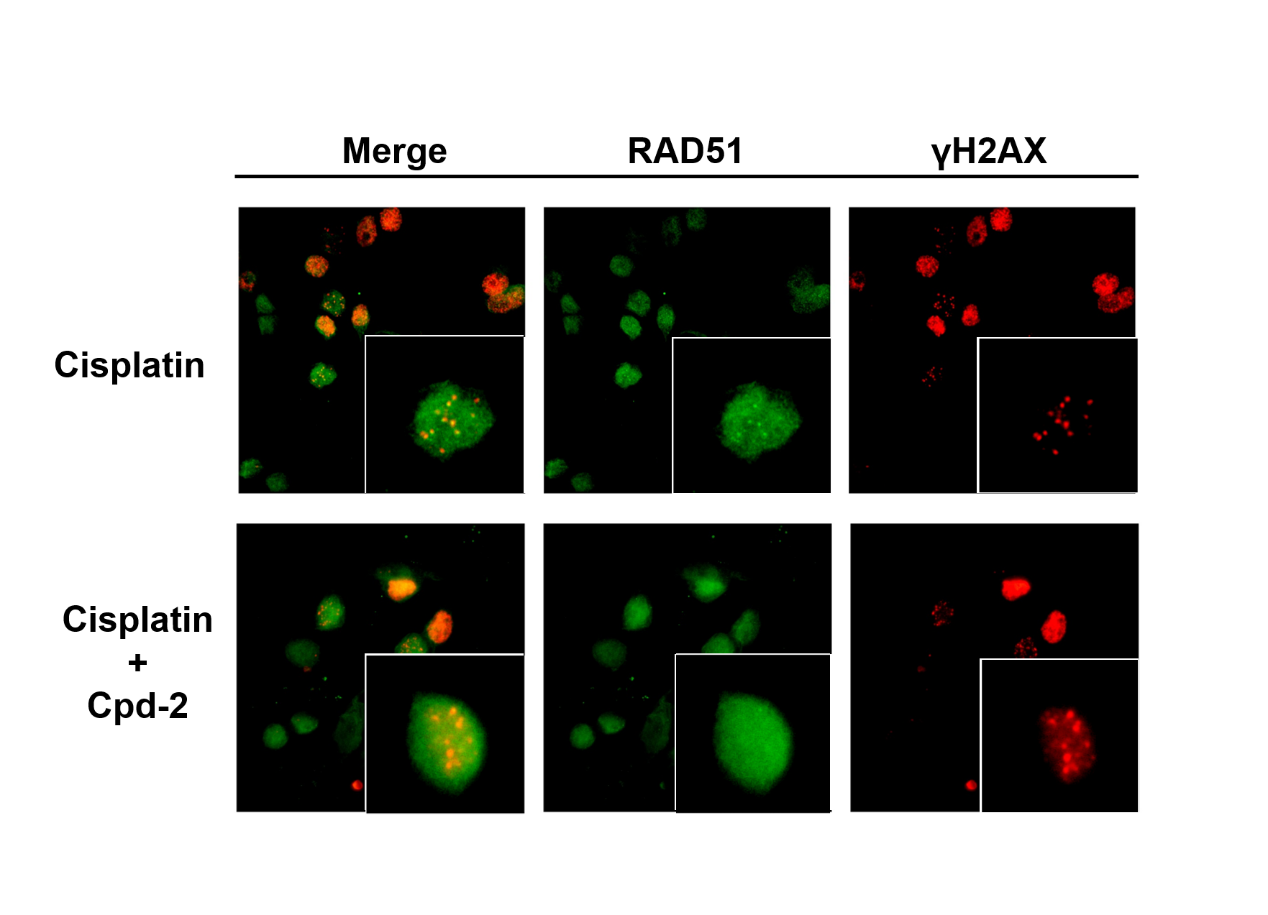
**

**Figure S1. RAD51 inhibitor inhibits foci formation in MDA-MB-468 cells.**

Image analysis of DNA damage and repair marker upon the compound treatment. MDA-MB-468 cells were treated with DMSO (upper panel) or Cpd-2 (10μM, lower panel) for 2 days, then cells were exposed to cisplatin (10μM) for 2 h (upper and lower panels) and stained 5 h later.


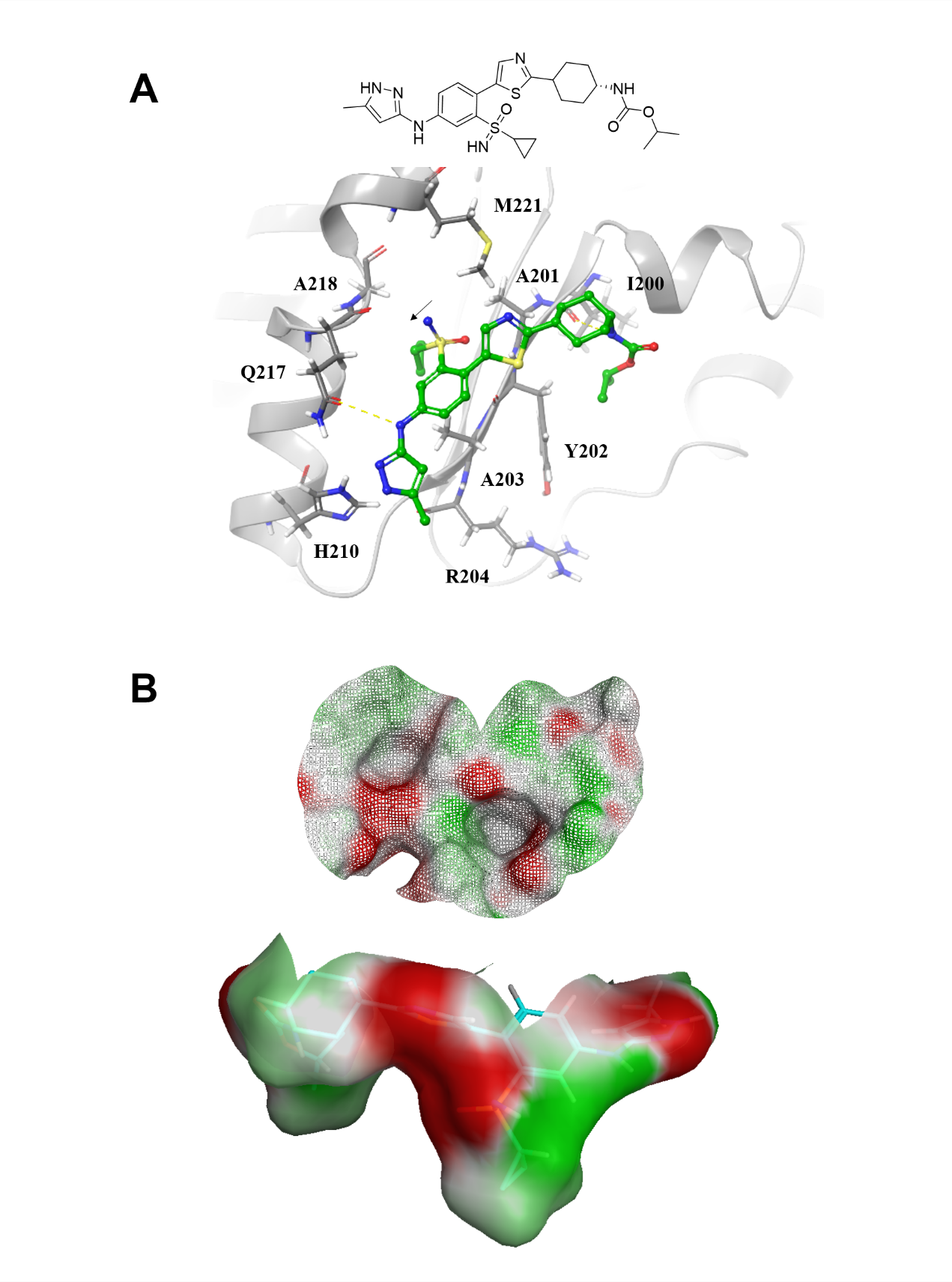


**Figure S2. Binding of RAD51 inhibitor to RAD51 protein.**

(A) The binding mode to RAD51 of Cpd-5.

(B) The analysis of the electrostatic potential of RAD51 pockets and Cpd-5.
